# Remote programming in stage I sacral neuromodulation: a multicentre prospective feasibility study

**DOI:** 10.1097/JS9.0000000000001140

**Published:** 2024-02-07

**Authors:** Jibo Jing, Lingfeng Meng, Yaoguang Zhang, Xinhao Wang, Wen Zhu, Qingwei Wang, Li Lu, Wei Song, Yifei Zhang, Yan Li, Jiaxing Ning, Haoran Wang

**Affiliations:** a Beijing Hospital, National Center of Gerontology, Institute of Geriatric Medicine, Chinese Academy of Medical Sciences & Peking Union Medical College; b Peking University Fifth School of Clinical Medicine, Beijing; c Department of Urology, The First Affiliated Hospital of Zhengzhou University, Zhengzhou; d Department of General Surgery, Sun Yat-sen University Sixth Affiliated Hospital, Guangzhou; e Department of Urology, Shandong Provincial Hospital; f Department of Urology, Qilu Hospital of Shandong University, Jinan; g Department of Urology, Institute of Urology, The First Affiliated Hospital of Anhui Medical University, Hefei, China

**Keywords:** lower urinary tract dysfunction, remote programming, sacral neuromodulation, stage I surgery

## Abstract

**Objective:**

Sacral neuromodulation (SNM) has emerged as an effective therapy for refractory lower urinary tract dysfunction (LUTD). Remote programming holds promise in addressing the time and economic burdens associated with outpatient programming, especially for patients in the observation period following Stage I implant surgery (where the lead is implanted first without the pulse generator). The study aimed to explore the effectiveness and patient satisfaction of remote programming for Stage I SNM patients, and analyze the benefits patients gain from remote programming.

**Methods:**

This prospective study was conducted at multiple high-level clinical SNM centres in China. Patients requiring SNM implantation were enroled and divided into two groups based on patient preference: remote programming (RP) group and outpatient control (OC) group. Patient attitudes toward RP were assessed through questionnaires, and the degree of symptom improvement was compared between the two groups to explore the usability of RP.

**Results:**

A total of 63 participants from 6 centres were included in the study, with 32 belonging to the RP group. The remote programming system presents a high level of usability (98%) and willingness (satisfaction rate: 96.83%) in result of questionnaire. RP showed a significant advantage in improving patients’ score of ICSI/ICPI (medianΔICSI/ICPI RP vs. OC= −13.50 vs −2, *P*=0.015). And slightly ameliorate urinary symptoms such as pain (medianΔVAS RP vs. OC= −1 vs 0, *P*= 0.164) and urgency (medianΔOBASS −2.5 vs. −1, *P*= 0.,229), but the difference was not statistically significant. RP did not significantly impact the quality of life of patients (*P*=0.113), so do the rate of phase-two conversion (*P*= 0.926) or programming parameters.

**Conclusion:**

To the best of our knowledge, the presented study is the first multicenter research focusing on the remote programming of Stage I SNM patients. Through the clinical implementation and patient feedback, we demonstrate that remote programming is not inferior to in-person programming in terms of success rate, effectiveness, safety, and patient satisfaction.

## Introduction

Highlights

- The presented research is the first multicenter clinical study of remote programming for patients post-stage I sacral neuromodulation surgery around the world.
- This study employed a prospective multicenter research approach to investigate patients’ willingness, user experience, and the effectiveness of remote control for sacral nerve modulation, as reflected by multiple objective indicators. It represents the outcomes of remote programming therapy in the Chinese population.

Refractory lower urinary tract dysfunction (LUTD) refers to non-obstructive functional abnormalities in the bladder and urethra during the storage and/or voiding phases, resulting from various neurogenic or non-neurogenic factors^1,2^. LUTD encompasses a wide spectrum of disorders, presenting with a range of symptoms, from continuous urinary leakage to urinary retention, recurrent urolithiasis, bladder pain, and recurrent urinary tract infections (UTIs). These conditions can ultimately lead to a significant decline in the quality of life and have a detrimental impact on the upper urinary tract, causing substantial patient distress and imposing a significant economic burden^3,4^.

Sacral neuromodulation (SNM) has a history of over thirty years in the treatment of refractory LUTD and was approved for clinical use by the U.S. Food and Drug Administration in 1997^5,6^ In China, SNM has been applied for nearly a decade, with recent developments and expansions^7,8^. While SNM can effectively improve the clinical symptoms of patients with refractory LUTD, patients often need to visit the hospital regularly for programming adjustments to maintain satisfactory efficacy.

The term “Stage I surgery” is commonly utilized in staged sacral nerve modulation therapy. In this procedure, a lead wire is initially implanted into the sacral foramen without the inclusion of a pulse generator. Subsequently, during the postoperative Stage I, patients often necessitate more frequent programming to ensure they attain the best therapeutic results.

Due to the fact that most qualified SNM centres in China are located in first-tier cities, and given the vast geographical expanse and uneven regional healthcare development in our country, many patients find it challenging to obtain timely and appropriate adjustments and planning after seeking medical treatment. In their pursuit of suitable programming, these patients often incur substantial time and economic costs travelling to hospitals with the implanted devices. Undoubtedly, it not only increases the time and economic burden on patients but also adversely affects their willingness to seek medical care and their medical compliance, ultimately leading to suboptimal long-term postoperative outcomes^9^.

Tsinghua University’s PINS Corporation has developed a novel SNM product, utilizing their unique remote programming technology. In simple terms, this technology allows patients to contact their physicians via the internet or mobile 4G/5G networks, from the comfort of their homes or any location with internet access, to adjust parameters and improve their symptoms. This technology undoubtedly alleviates many of the aforementioned challenges, and prior research by our team suggests that patients have benefited from it^10^. However, whether Stage I patients can benefit from remote control and whether it is beneficial for the conversion to Stage II remains a subject for further investigation.

To the best of our knowledge, the presented study is the first multicenter research focusing on the remote programming of Stage I SNM patients, aims to explore the benefits of remote programing for patients after Stage I SNM surgery, with the goal of guiding the refinement of remote programing technology.

## Method

**Figure 1:**
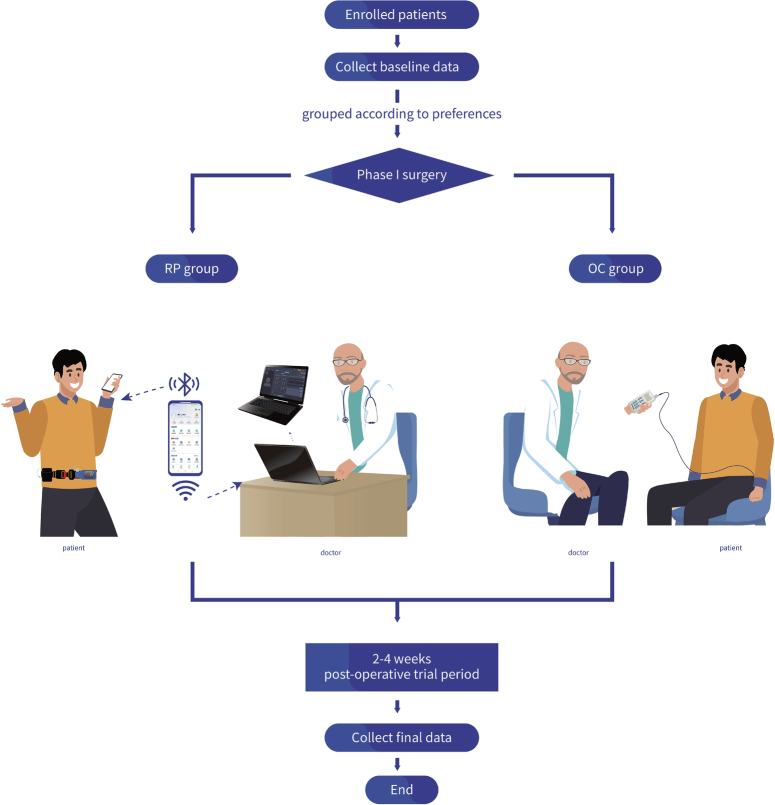
Study design. OC, outpatient control; RP, remote programming.

The presented study is a prospective multicenter case-control research and has been reported in line with the STROCSS criteria^11^, Supplemental Digital Content 1, http://links.lww.com/JS9/B825. Enroled patients are categorized into either the Remote Programing Group (RP group) or the Outpatient Control Group (OC group) based on their preferences. Initially, baseline research data is collected from the patients, followed by the surgical procedure. After the completion of the patients’ Stage I trial period (typically spanning 2–4 weeks), a second set of research data is collected. Subsequently, statistical analysis is conducted to explore the therapeutic effects, economic benefits, and analyze potential risk factors among the patients (Fig. 1).

### Patient information

This study was conducted across six accredited clinical centres specializing in SNM. The participants were patients who met the diagnostic criteria for refractory LUTD and willingly participated in the study after providing informed consent.

### Inclusion criteria were as follows

1. Age older than or equal to 18 years;
2. Patients implanted with sacral nerve stimulation systems due to refractory lower urinary tract dysfunction between August 2022 and June 2023, such as overactive bladder, neurogenic bladder, interstitial cystitis/chronic pelvic pain syndrome, non-obstructive urinary retention, etc.;
3. Agreement to refrain from arbitrarily altering medications that may affect lower urinary tract symptoms and pelvic function during the trial period;
4. Voluntary participation in the research study and signing of informed consent;
5. Possess the needed ability to communicate effectively with the investigators and willingness to adhere to the requirements of the entire trial.
6. Exclusion criteria included:
7. Pregnant or lactating women;
8. Concurrent uncontrolled urinary tract infections, urinary tract obstruction, urological tumours;
9. Mental health conditions hindering collaboration with medical professionals;
10. Presence of severe concomitant diseases, such as non-urological malignant tumours, significantly impacting health;
11. Other circumstances deemed unsuitable for participation in the study by the investigators.

### Clinical data

Data collection primarily comprises two components: a self-designed questionnaire and symptom questionnaires. The self-designed questionnaire was developed by the researchers to supplement remote control information in alignment with the practical aspects of the study. The questionnaire includes information about the time and economic costs (including costs incurred by accompanying family members) associated with each routine hospital visit for programming, reasons for requesting remote programming, the anticipated frequency of remote control, the extent to which remote control has been beneficial for the patients, the ease of operating remote control, patient satisfaction with remote control, and their likelihood of recommending it, among other aspects.

The symptom questionnaire focuses on specific aspects of the patients’ Lower Urinary Tract Dysfunction (LUTD) and primarily includes the following: Standard voiding diary; Visual analogue scale (VAS); Overactive Bladder Symptom Score (OABSS); Voiding Symptom Quality of Life Questionnaire (QoL); Interstitial Cystitis O’Leary-Sant Symptom and Problem Index (ICPI&ICSI); The scales used in the research are available in Supplement 1 for reference.

### Remote programming system

The remote programming system, refers to a programming method distinct from traditional face-to-face programming. Specifically, it involves utilizing specialized remote devices to communicate and conduct programming and debugging with physicians or device engineers through wireless or wired network connections.

**Figure 2:**
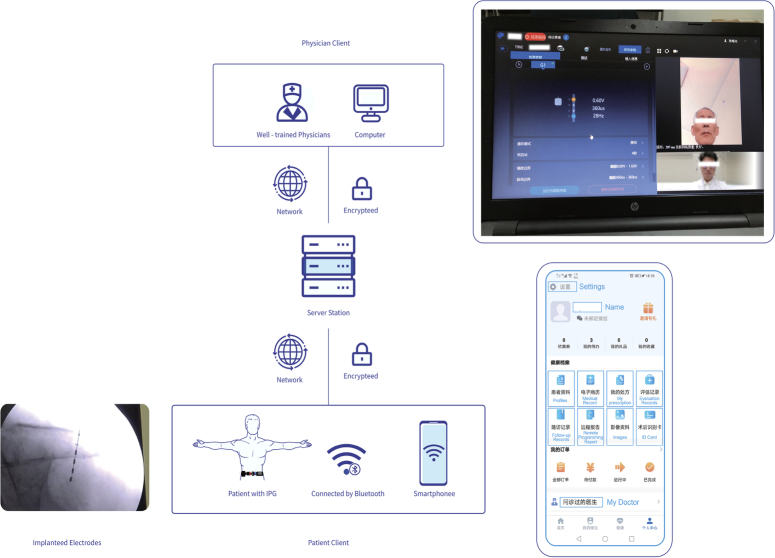
Schematic diagram of how remote programming system works.

The remote programming system adopted by the presented was developed and launched by Tsinghua Pins Medical Technology Co., Ltd. in 2019, is currently applicable in several neuromodulation system^10,12,13^. The system comprises three main components: the Doctor’s Console, the Patient’s Interface, and the Central Server terminal. The patient’s interface and the doctor’s console are connected to the central server through two different versions of applications (Fig. 2).

In our remote programming system, patients at home use their mobile devices (tablets or smartphones) to access the patient version of the application. They can schedule appointments with their doctors and pair their devices with the implanted pulse generator (IPG) via Bluetooth for remote programming. Video, audio, and stimulation signals are transmitted to the central server.

Meanwhile, in the physician’s interface doctors are able to adjust the patient’s stimulation program based on their condition. The principle involves doctors sending programming commands to the central server through the interface, and the server converts these commands into operational instructions recognizable by the stimulator, pushing them to the patient’s interface. This facilitates functions such as audio-visual communication, telemetry, parameter adjustments, electrode impedance detection, and more. Research by Chinese scholars has confirmed that this technology is just as effective as in-person programming in outpatient settings^14^.

Furthermore, to ensure data security, we have three levels of protection: secure Hyper Text Transfer Protocol over Secure Socket Layer (HTTPS) with asymmetric encryption, secure Low Energy Bluetooth (BLE), and Near Field Communication (NFC) mode for device communication. The patient’s app includes a “one-click restore/shutdown” function for network issues.

Figure 2 illustrates the remote programming system, which consists of three parts: the patient’s interface, the physician’s interface, and the central server. The physician’s interface is depicted in the lower right corner, and the patient’s interface is shown in the upper right corner.

### Ethics

This study has undergone ethical review at Beijing Hospital and the various research unit subcenters, with an ethics approval number of 2020BJYYEC07802. This approval ensures the ethical compliance of the study and underscores the ethical standards that researchers are obligated to adhere to. Furthermore, this study is registered with the China Clinical Trial Center, with registration number ChiCTR2000036677. (https://www.chictr.org.cn/index.html).

Prior to the commencement of this study, each participant undergoes a comprehensive informed consent procedure. The study will maintain transparency at all times, and the research plan and results will be publicly disclosed. We employ institutional-level review oversight to prevent potential conflicts of interest, ensuring the fairness and reliability of the study.

### Statistical analysis

Initially, data cleaning was conducted to ensure data quality and consistency. Descriptive statistical methods were used to analyze baseline data to understand the characteristics of the two patient groups. For continuous variables that followed a normal distribution, mean and standard deviation were presented, and *t*-tests were used for comparisons. For variables that did not follow a normal distribution, median and interquartile range were presented, and the Mann–Whitney U test was used for group comparisons. Categorical variables were presented as counts (percentages), and χ^2^ tests were used for between-group comparisons.

Subgroup analyses, if required, would be conducted to delve deeper into the differences in treatment outcomes and economic benefits among different subgroups of patients. All statistical analyses would be performed using IBM SPSS Statistics 19.0.0.329, and visualizing by GraphPad Prism 8.3.0, Python to ensure the accuracy and reliability of the results.

## Result

### General information

**Figure 3:**
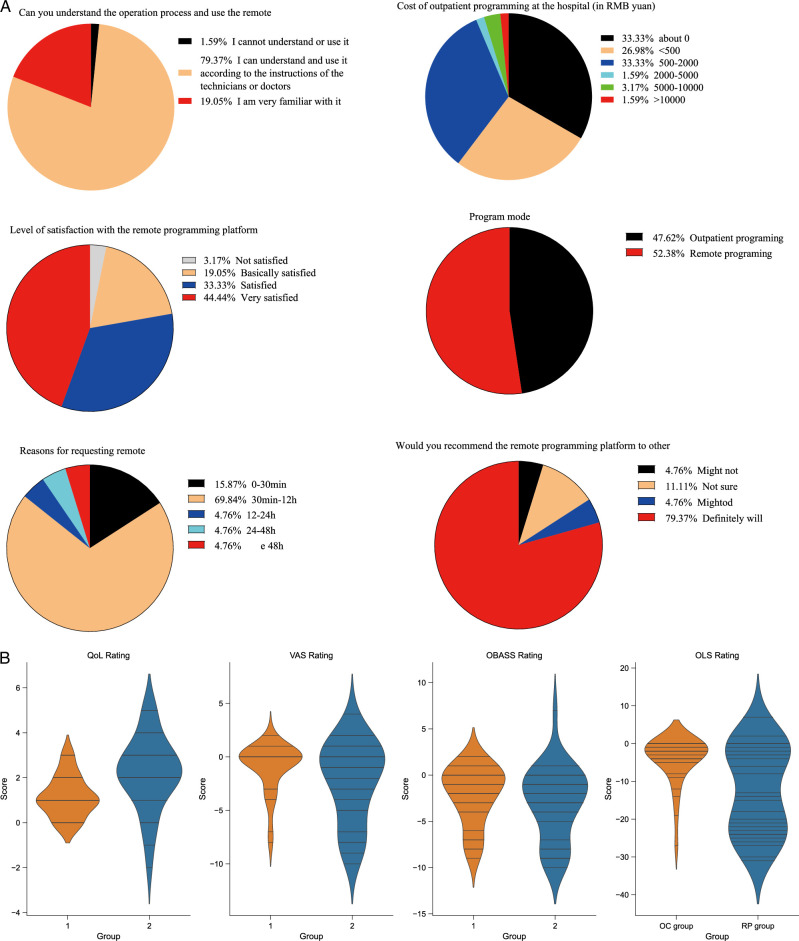
Statistical graphs of self-designed questionnaire and symptom questionnaire results. A: Pie chart of self-designed questionnaire survey results; B: Violin plot comparing quality of life scores (QOL), visual analog scale for pain scores (VAS), overactive bladder symptom scores (OBASS), and International O'Leary-Sant Symptom and Problem Index (OLS) between the RP group and OC group. OBASS, Overactive Bladder Awareness and Symptom Score; OC, outpatient control; QoL, Voiding Symptom Quality of Life Questionnaire; P, remote programming; VAS, visual analogue scale.

A total of 63 participants were included in the study across 6 centres. Among them, 32 belonged to the RP group. The patients were primarily from North China, East China, and Central China. The vast majority of patients decided on surgery due to difficulties in urination caused by neurogenic lower urinary tract dysfunction. Other symptoms include overflow urinary incontinence, pain or dysuria (painful urination), frequent urination, and urinary retention. In most cases, the modulating electrodes were implanted in the left S3 sacral foramen (about 56%). There were no significant differences between the two groups in terms of general data such as age and sex. However, it’s noteworthy that there were statistically significant differences in diagnoses and some symptoms between the two groups. Specifically, 7 patients with interstitial cystitis (IC) were included in the RP group. Measures will be taken to avoid the deviation of statistical analysis from reality in subsequent analysis. (Table 1, Fig. 3A).

**Table 1:** General Information of enroled patients.

	RP group (*n*=32)	OC group (*n*=31)	*t*/χ^2^	*p*
Age, mean (SD)	44.75 (20.28)	51.10 (17.08)	1.341	0.185
Sex			2.045	0.153
Male	16	21		
Female	16	10		
Implant side			3.625	0.161
Left	18	18		
Right	14	10		
Double	0	3		
Implanted sacral hiatus			2.506^a^	0.113
S3	32	27		
S4	0	4		
Residence type			0.762	0.383
Countryside	12	16		
City	20	20		
Diagnose			7.653	0.022
NLUTD	24	30		
IC	7	0		
UUI/OAB	1	1		
Difficulty urinating			5.028	0.025
Do not have	12	4		
Have	20	27		
Overflow urinary incontinence			0.169	0.681
Do not have	28	26		
Have	4	5		
Pain or dysuria			3.059	0.08
Do not have	25	29		
Have	7	2		
Frequent urination			3.65	0.056
Do not have	20	26		
Have	12	5		
Urgency with incontinence			7.497	0.006
Do not have	21	29		
Have	11	2		
Urinary retention			5.341	0.021
Do not have	27	18		
Have	5	13		

IC, interstitial cystitis; NLUTD, neurogenic lower urinary tract dysfunction; OAB, overactive bladder; OC, outpatient control; RP, remote programming; UUI, Urge Urinary Incontinence.

a Using χ^2^ test with continuity correction.

### Survey results

Through the survey, the study found that although the cost of a single session of remote programming is relatively low for most patients (~60% of patients have a single-session cost <500 yuan), the time cost is quite high, with the majority needing to spend more than a working day. Therefore, upon group comparison, it becomes evident that the economic cost associated with outpatient control (OC) group is notably lower (*P*=0.02), possibly explaining their preference for choosing this group when enroling. It’s also noteworthy that the two groups differ in their motivations for seeking remote programming. The Remote Programming (RP) group tends to opt for it more due to considerations of time and economic costs, while OC patients primarily lean towards remote programming because of the frequent recurrence of symptoms (*P*=0.02). No substantial differences were observed between the two groups in response to other questionnaire items. In fact, although over 80% of patients stated that their desired frequency of programming is not fixed and occurs only when needed, the majority had a frequency greater than once a week during the observation period. This was even more true for the RP (remote programming) group, indicating that the actual demand for programming is greater than what patients themselves think. In summary, the remote programming system presents a high level of usability (98%) and willingness (satisfaction rate: 96.83%) in result of questionnaire. (Table 2)

**Table 2:** The result of self-made survey.

		RP group (*n*=32)		OC group (*n*=31)			
Item	Category	*n*	%	*n*	%	χ^2^	*p*
Cost of outpatient programming at the hospital (in RMB yuan)	about 0	9	28.13	12	38.71	13.35	0.02
	<500	5	15.63	12	38.71		
	500–2000	17	53.13	4	12.90		
	2000–5000	0	0.00	1	3.23		
	5000–10 000	1	3.13	1	3.23		
	>10 000	0	0.00	1	3.23		
Duration of outpatient programming at the hospital	0–30 min	7	21.88	3	9.68	3.40	0.49
	30 min–12 h	19	59.38	25	80.65		
	12–24 h	2	6.25	1	3.23		
	24–48 h	2	6.25	1	3.23		
	≥48 h	2	6.25	1	3.23		
Reasons for requesting remote programming (multiple choices)	Regular follow-up	6	18.75	6	19.35	10.40	0.02
	Symptoms recurring/no improvement	12	37.50	17	54.84		
	Seeking further improvement	14	43.75	4	12.90		
	Discomfort or side effects	0	0.00	4	12.90		
Frequency of programming	Less than once a week	1	3.13	1	3.23	8.95	0.06
	Once a week	6	18.75	0	0.00		
	Once per 1–3 week	1	3.13	1	3.23		
	A month or more	2	6.25	0	0.00		
	Not fixed, as needed	22	68.75	29	93.55		
Reasons for choosing remote programming (multiple choices)	Professional, expert-level service	14	43.75	10	32.26	0.61	0.43
	Convenient for adjustments	18	56.25	23	74.19	1.51	0.22
	Time and cost-saving	23	71.88	23	74.19	0.00	1.00
	Improved effectiveness	11	34.38	9	29.03	0.03	0.85
	Reduced travel due to pandemic reasons	10	31.25	8	25.81	0.04	0.84
Can you understand the operation process and use the remote	I cannot understand or use it	1	3.13	0	0.00	2.64	0.27
	I can understand and use it according to the instructions	27	84.38	23	74.19		
	I am very familiar with it	4	12.50	8	25.81		
Level of satisfaction with the remote programming platform	Not satisfied	2	6.25	0	0.00	6.32	0.10
	Basically satisfied	9	28.13	3	9.68		
	Satisfied	8	25.00	13	41.94		
	Very satisfied	13	40.63	15	48.39		
Would you recommend the remote programming platform to other	Might not	3	9.38	0	0.00	7.35	0.06
	Not sure	5	15.63	2	6.45		
	Might do	0	0.00	3	9.68		
	Definitely will	24	75.00	26	83.87		

OC, outpatient control; RP, remote programming.

### Baseline symptoms

Through the standard urination diary, researchers have recorded the basic urination conditions of patients before surgery. The results showed that the median daily urination frequency is around seven times per day, and the median volume per urination is 170 ml. The median OBASS is 4–5 points, while the median QoL remains at around 2–3 points. During the baseline period, there was no statistically significant difference between the two groups in the above data. Interestingly, the baseline ICSI/ICPI scores for the RP group seemed higher than the OC group (median values 21.5 vs. 11 *P*=0.001). (supplement Table 1, Supplemental Digital Content 1, http://links.lww.com/JS9/B826).

### Symptom improvement

Symptom score scales were collected again from each patient at the end of the observation period (Supplementary Table 1, Supplemental Digital Content 1, http://links.lww.com/JS9/B826). Due to the difference in ICSI/ICPI between the two groups at baseline, the researchers decided to investigate the relationship between programmed mode and patient symptom improvement by examining the changes in the score scales before and after the observation period. The findings indicated a marginal enhancement in urinary symptoms, particularly in pain (median ΔVAS RP vs. OC=−1 vs. 0, *P*=0.164) and urgency (median ΔOBASS −2.5 vs. −1, *P*=0.229). Despite this, it’s essential to note that these disparities did not attain statistical significance. And RP did not significantly impact the quality of life of patients (*P*=0.113) (Table 3). On the other hand, there was a more notable improvement in ICSI/ICPI in the RP group (median −2 vs. −13.5, *P*=0.015). We wondered if this was because the RP group included 7 IC patients. To this end, we conducted a subgroup analysis. The results showed that after excluding IC patients, there was no significant difference between the two groups at baseline(median ICSI/ICPI RP vs. OC=5 vs. 3, *P*=0.07) and in the change values(median ΔICSI/ICPI RP vs. OC=5 vs. 6, *P*=0.70) (Supplementary Table 2, Supplemental Digital Content 1, http://links.lww.com/JS9/B826, 4, Supplemental Digital Content 1, http://links.lww.com/JS9/B8, 5, Supplemental Digital Content 1, http://links.lww.com/JS9/B826), which confirmed our assumption.

**Table 3:** The change value of certain urinary indices before and after the observational period.

	OC group (*n*=31)			RP group (*n*=32)				
	Median	q1	q3	Median	q1	q3	Z	p
Average urination frequency per day	0.000	−4.000	2.000	−2.500	−9.500	0.000	−1.862	0.063
Average urine volume per void	63.000	10.000	90.000	34.000	11.500	83.750	−0.674	0.500
Urgency score	0.000	−2.000	0.000	−1.000	−2.000	0.000	−0.678	0.498
Average incontinence episodes	0.000	−1.000	0.000	0.000	−0.875	0.000	−0.408	0.683
Average incontinence volume	0.000	−3.000	0.000	0.000	0.000	0.000	−0.664	0.507
VAS	0.000	0.000	0.000	−1.000	−5.000	0.000	−1.391	0.164
QoL	2.000	1.000	3.000	1.500	1.000	2.000	−1.010	0.313
OBASS	−1.000	−4.000	0.000	−2.500	−6.500	0.000	−1.204	0.229
ICSI/ICPI	−2.000	−5.000	0.000	−13.50	−23.75	−0.250	−2.428	0.015

ICSI/ICPI, Interstitial Cystitis O’Leary-Sant Symptom and Problem Index; OBASS, Overactive Bladder Awareness and Symptom Score; OC, outpatient control; QoL, Voiding Symptom Quality of Life Questionnaire; RP, remote programming; VAS, visual analogue scale.

### Final

Upon completion of the observation period, we collected pertinent patient-controlled data. The median observation period for both groups of patients is 28 days, with no significant differences(*P*=0.861). The data indicate that the voltage utilized in the OC group was slightly higher (*P*=0.04), whereas there were no significant differences in terms of pulse interval and frequency. Furthermore, there were no noticeable differences between the two groups in terms of phase-two conversion rate (*P*= 0.926), suggesting that remote patient-controlled interventions possess conversion capabilities akin to those in outpatient settings. (Supplementary Table 3, Supplemental Digital Content 1, http://links.lww.com/JS9/B826)

## Discussion

Sacral nerve modulation therapy, as a first-line treatment for refractory neurogenic lower urinary tract dysfunction, has gained widespread promotion and application worldwide over the past decade, bringing significant clinical benefits to a large number of patients^15–17^. However, conventional sacral nerve modulation therapy is primarily conducted through in-person programming, requiring patients to visit a healthcare facility for each adjustment. In China, most centres with authoritative qualifications for sacral nerve modulation therapy are located in major eastern cities, which imposes a significant financial burden on many patients suffering from the condition. This, to some extent, reduces their willingness to undergo in-person programming.

Currently, there have been global efforts to implement remote programming for sacral neuromodulation therapy. However, these studies predominantly focus on patients after permanent implant and are often retrospective in nature^14,18,19^. Concerning SNM patients after Stage I surgery, while foundational research has explored remote programming techniques, there’s a significant absence of clinical studies reporting on the remote programming for this specific patient cohort. To the best of our knowledge, the presented study is the first multicenter research focusing on the remote programming of Stage I SNM patients. In our team’s previous research, we found that remote programming might help address the aforementioned problem^10^. However, most of these studies were retrospective, and as far as we know, patients receiving sacral nerve modulation therapy are predominantly those with refractory lower urinary tract dysfunction, often accompanied by intense subjective symptoms such as voiding difficulty, urinary frequency, and pain. Prior research has shown significant discrepancies between retrospective subjective data and real-time semi-objective data, likely due to patients’ tendency to embellish or exaggerate their recollections^20,21^. Additionally, previous studies mainly focused on patients with permanent implants who require fewer programming adjustments, making remote programming less beneficial^10^. Therefore, we designed a prospective case-control study, focusing on patients in the observation period following stage I implantation surgery, who frequently require programming adjustments to find suitable parameters. This study explores whether they can benefit from remote programming, and to our knowledge, it represents the first prospective multicenter clinical study in this regard.

Based on preliminary research, the study included 63 participants and six participating centres, which are primarily distributed in eastern, central, and northern China, representing the highest level of sacral nerve modulation therapy in the country. This ensures that patients get the right level of treatment and accurately reflects the diversity of the patient population. Our results indicate that the included patients are primarily those with neurogenic lower urinary tract dysfunction (NLUTD), IC, and overactive bladder (OAB), with NLUTD being the most prevalent. This contrasts with what was observed in our previous studies^9^. We speculate that this difference may be attributed to the fact that patients willing to participate in the study may generally have more severe symptoms compared to other populations, thereby altering the distribution of the diseases. It could also be a result of the relatively small sample size. We will endeavour to explore this issue further in future research studies.

From a results perspective, remote programming achieved similar efficacy and user experience as in-person programming. Both important subjective symptom assessment scales and semi-objective voiding diaries showed similar changes between the two groups. Notably, patients in the remote programming group exhibited a more pronounced decrease in ICSI/ICPI scores. Further analysis revealed that this was mainly due to the presence of seven patients with IC in the RP group. Unfortunately, IC patients were not included in the OC group to explore the effects of RP on this specific population. However, upon analyzing the indicators in the RP group specifically among patients with IC, we observed that despite IC patients showing a greater degree of improvement in indicators compared to patients with other diagnoses in the same group, the comparison of efficacy indicators between the RP and OC groups, after excluding IC patients, still leads to similar conclusions. However, through a comparison with retrospective data from IC patients receiving conventional programming, we found that IC patients using remote programming may experience greater symptom improvement, suggesting that IC patients may have a higher programming demand.

In other results, our questionnaire survey showed that a significant proportion came from rural areas. And the survey also indicated that most patients chose to undergo sacral nerve modulation therapy due to refractory NLUTD, aligning with previous research^10^. The economic cost of programming for most patients was not as high as initially imagined, but the time cost was substantial, underscoring the significant practical value of remote programming. Based on the experience of the remote programming system, the majority of participants reported that the remote system was a safe and satisfactory solution, worthy of wider adoption.

Additionally, technology is also one of the crucial factors influencing patients’ choice of remote programming. The remote programming system used in this study is derived from the classical face-to-face programming technology. Therefore, the two programming technologies show no significant differences in daily portability and usage. (Supplement Table 6, Supplemental Digital Content 1, Supplemental Digital Content 1, http://links.lww.com/JS9/B826) However, compared to clinic-based programming, remote programming requires a wired or wireless network technically, and patients have additional tasks: (1) using the patient-side programming app and (2) precisely describing their sensations regarding programming. Our survey results indicate that the majority of patients can use this system with guidance.

Nevertheless, it’s important to note that there is still a very small percentage of patients facing accessibility issues with remote programming due to technical reasons. We look forward to further improvements in the future. Nevertheless, our study still has room for improvement. Firstly, IC patients were not included in the OC group, and while we supplemented retrospective data for comparison, there may still be some bias. Secondly, despite addressing issues such as the lack of comparison with traditional programming and the absence of before-and-after symptom improvement comparisons compared to our previous study, the current research only collected subjective data such as symptom questionnaires. Since this study is mainly observational, we did not conduct invasive examinations like urodynamic studies before and after patient treatment. However, the study has demonstrated that remote programming is not inferior to in-person programming. In future research, if patients require urodynamic studies, we will conduct more in-depth exploration of the objective effects of remote programming.

Furthermore, it is important to note that this study primarily focused on the effectiveness of remote teleoperation, assessed through user satisfaction. Detailed comparative research on the technical aspects of remote teleoperation was not conducted, potentially limiting the broader application and further exploration of this technology. We acknowledge this limitation and plan to undertake more in-depth investigations in the future.

For similar reasons, real-time recording of all remote teleoperation parameters, such as feeling points and voltage, during the entire patient trial period was not implemented in this study. Only the final parameters were collected. While this may have limitations, the outcomes could prove beneficial for the clinical application of remote teleoperation.

In an upcoming study, soon to be published, we have summarized the parameter characteristics used by all patients transitioning to the second phase. We hope that this compilation will contribute to the wider adoption of this technology. Our commitment to advancing this research remains unwavering, and we plan to continually progress in this field.

## Conclusion

Through clinical implementation and patient feedback, this study formally demonstrates that remote programming is not inferior to in-person programming in terms of success rate, effectiveness, safety, and patient satisfaction. It supports the significant potential for the development and application of remote programming services for patients after Stage I surgery, which can provide efficient, cost-effective, and convenient programming services for a broader range of sacral nerve modulation patients.

## Ethical considerations

This study has undergone ethical review at Beijing Hospital and the various research unit subcenters, with an ethics approval number of 2020BJYYEC07802. Informed consent will be obtained from all study participants.

## Consent

Written informed consent has been obtained from the patient for the publication of this research and accompanying images. A copy of this written consent is available for review upon request by the Editor-in-Chief of the journal.

## Sources of funding

This research is supported by the National High-Level Hospital Clinical Research Funding (BJ-2021- 184 and BJ-2023-099).

## Author contribution

J.J. contributed to the project by performing writing, formal analysis, investigation, and data curation. M.L. played a role in conceptualization, project administration, and securing funding for the research. Z.Y. contributed to the project by providing supervision and expertise in methodology. W.X. was involved in investigation and validation of the research. Z.W. participated in the investigation and provided necessary resources. W.Q. contributed to the project by providing supervision and guidance. L.L. played a role in the investigation and contributed to the allocation of resources. S.W. was involved in the investigation and provided necessary resources. Z.Y. contributed to the investigation and provided resources for the project. L.Y. participated in the investigation and allocation of resources. N.J. was involved in the investigation phase of the project. W.H. participated in the investigation phase of the project. All authors declare that this manuscript is original, has not been published before and is not currently being considered for publication elsewhere. We confirm that the manuscript has been read and approved by all named authors and that there are no other persons who satisfied the criteria for authorship but are not listed. We further confirm that the order of authors listed in the manuscript has been approved by all of us. We understand that the Corresponding Author is the sole contact for the Editorial process. He/she is responsible for communicating with the other authors about progress, submissions of revisions and final approval of proofs.

## Conflicts of interest disclosure

The authors declare that there is no conflict of interest to disclose.

## Research registration unique identifying number (UIN)

This study is registered with the China Clinical Trial Center, with registration number ChiCTR2000036677.

## Guarantor

Zhang Yaoguang.

## Data collection

Data were collected from [patients, medical records, surveys] between 2022.08.01] and [2023.06.30]. Data will include information on [demographics, medical history, exposure variables, Lower Urinary Tract Dysfunction (LUTD) and primarily includes the following: Standard voiding diary; Visual analogue scale (VAS); Overactive Bladder Symptom Score (OABSS); Voiding Symptom Quality of Life Questionnaire (QoL); Interstitial Cystitis O’Leary-Sant Symptom and Problem Index (ICPI&ICSI)].

## Data sources: study population

The study population were patients who met the diagnostic criteria for refractory Lower Urinary Tract Dysfunction (LUTD) and willingly participated in the study after providing informed consent.

## Data handling and management

Data will be collected and managed using [Excel]. All data will be stored securely to maintain confidentiality and privacy.

## Data analysis

Data analysis will be performed using IBM SPSS Statistics 19.0.0.329. Statistical tests and methods will include [Descriptive statistical methods were used to analyze baseline data to understand the characteristics of the two patient groups. For continuous variables that followed a normal distribution, mean and standard deviation were presented, and *t*-tests were used for comparisons. For variables that did not follow a normal distribution, median and interquartile range were presented, and the Mann–Whitney U test was used for group comparisons. Categorical variables were presented as counts (percentages), and χ^2^ tests were used for between-group comparisons.

## Data sharing

The data used to support the findings of this study are available upon request from the corresponding author. The data are not publicly available due to privacy and ethical considerations. Requests for access to the data should be directed to corresponding author.

## Data statement last updated:

This data statement was last updated on [2023.08.31].

## Data availability statement

The data used to support the findings of this study are available upon request from the corresponding author. The data are not publicly available due to privacy and ethical considerations. Requests for access to the data should be directed to corresponding author.

## Provenance and peer review

Not commissioned, externally peer-reviewed.

## Supplementary Material

SUPPLEMENTARY MATERIAL
