# Supplementary material for: Remote programming in stage I sacral neuromodulation: a multicentre prospective feasibility study
Source: Int J Surg. 2024 Feb 7;110(4):2104–14. doi: 10.1097/JS9.0000000000001140 (PMC11020101; doi:10.1097/JS9.0000000000001140)
Supplement: SUPPLEMENTARY MATERIAL [file js9-110-2104-s002.docx]

Supplemental table 1. The value of certain urinary indices before the surgery

|  | OC group(n=31) | | | RP group(n=32) | | | Z | p |
| --- | --- | --- | --- | --- | --- | --- | --- | --- |
|  | median | q1 | q3 | median | q1 | q3 |  |  |
| Average urination frequency per day | 6.00 | 5.00 | 12.50 | 12.00 | 7.63 | 18.75 | -2.52 | 0.01 |
| Average urine volume per void | 112.00 | 50.00 | 200.00 | 101.50 | 58.00 | 169.75 | -0.12 | 0.90 |
| Urgency score | 2.00 | 0.00 | 3.00 | 2.00 | 0.00 | 4.00 | -0.70 | 0.48 |
| Average incontinence episodes | 0.00 | 0.00 | 2.50 | 0.00 | 0.00 | 1.88 | -0.37 | 0.71 |
| Average incontinence volume | 0.00 | 0.00 | 15.00 | 0.00 | 0.00 | 17.50 | -0.91 | 0.36 |
| VAS | 0.00 | 0.00 | 3.00 | 3.00 | 0.00 | 7.25 | -1.98 | 0.05 |
| QoL | 3.00 | 1.00 | 3.00 | 2.00 | 1.00 | 3.00 | -0.83 | 0.41 |
| OBASS | 4.00 | 1.00 | 11.00 | 5.00 | 2.50 | 9.00 | -0.85 | 0.40 |
| ICSI/ICPI | 11.00 | 3.00 | 15.00 | 21.50 | 7.00 | 28.75 | -2.91 | 0.00 |

Supplemental table 2. The value of certain urinary indices after the observational period

|  | OC group(n=31) | | | RP group(n=32) | | | Z | p |
| --- | --- | --- | --- | --- | --- | --- | --- | --- |
|  | median | q1 | q3 | median | q1 | q3 |  |  |
| Average urination frequency per day | 7.00 | 5.00 | 8.00 | 8.00 | 6.00 | 10.75 | -1.45 | 0.15 |
| Average urine volume per void | 170.00 | 125.00 | 215.00 | 184.50 | 108.50 | 255.25 | -0.31 | 0.76 |
| Urgency score | 1.00 | 0.00 | 2.00 | 1.00 | 0.00 | 2.00 | -0.16 | 0.87 |
| Average incontinence episodes | 0.00 | 0.00 | 2.00 | 0.00 | 0.00 | 0.00 | -2.85 | 0.00 |
| Average incontinence volume | 0.00 | 0.00 | 12.00 | 0.00 | 0.00 | 0.00 | -2.78 | 0.01 |
| VAS | 0.00 | 0.00 | 1.00 | 0.00 | 0.00 | 2.00 | -1.34 | 0.18 |
| QoL | 5.00 | 4.00 | 5.00 | 4.00 | 3.00 | 4.00 | -3.15 | 0.00 |
| OBASS | 2.00 | 1.00 | 5.00 | 1.50 | 1.00 | 5.00 | -0.73 | 0.47 |
| ICSI/ICPI | 6.00 | 1.00 | 9.00 | 5.00 | 0.25 | 10.50 | -0.25 | 0.80 |

Supplemental Table 3. The final parameters of enrolled patients.

|  | RP group(n=32) | | | OC group(n=31) | | | Z/χ2 | p |
| --- | --- | --- | --- | --- | --- | --- | --- | --- |
|  | median | q1 | q3 | median | q1 | a3 |  |  |
| **Magnitude（V）** | 1.400 | 0.913 | 2.038 | 2.000 | 1.100 | 3.200 | -2.100 | 0.036 |
| **Pulse Interval (μs)** | 210.000 | 210.000 | 210.000 | 210.000 | 210.000 | 210.000 | -1.303 | 0.192 |
| **Frequency (Hz)** | 17.500 | 14.000 | 40.000 | 20.000 | 14.000 | 40.000 | -0.930 | 0.352 |
| **Testing duration** | 28 | 26.5 | 29.5 | 28 | 21 | 32 | -0.175 | 0.861 |
| **Whether to undergo permanent surgery** | | | | | | | | |
| yes | 21.000 | | | 20.000 | | | 0.009 | 0.926 |
| no | 11.000 | | | 11.000 | | |  |  |

Supplemental table 4. The value of certain urinary indices before the surgery（exclude IC）

|  | OC group(n=31) | | | RP group(n=25) | | | Z | p |
| --- | --- | --- | --- | --- | --- | --- | --- | --- |
|  | median | q1 | q3 | median | q1 | q3 |  |  |
| Average urination frequency per day | 6.00 | 5.00 | 12.50 | 11 | 6.5 | 17 | -1.84 | 0.07 |
| Average urine volume per void | 112.00 | 50.00 | 200.00 | 105 | 58 | 164.5 | -0.08 | 0.93 |
| Urgency score | 2.00 | 0.00 | 3.00 | 1 | 0 | 4 | -0.11 | 0.91 |
| Average incontinence episodes | 0.00 | 0.00 | 2.50 | 0 | 0 | 2 | -0.11 | 0.91 |
| Average incontinence volume | 0.00 | 0.00 | 15.00 | 0 | 0 | 20 | -0.26 | 0.80 |
| VAS | 0.00 | 0.00 | 3.00 | 0 | 0 | 4 | -0.89 | 0.37 |
| QoL | 3.00 | 1.00 | 3.00 | 2 | 1 | 3 | -1.14 | 0.25 |
| OBASS | 4.00 | 1.00 | 11.00 | 5 | 2 | 8 | -0.61 | 0.54 |
| ICSI/ICPI | 11.00 | 3.00 | 15.00 | 14 | 5 | 24 | -1.81 | 0.08 |

Supplemental table 5. The value of certain urinary indices after the observational period (exclude IC)

|  | OC group(n=31) | | | RP group(n=25) | | | Z | p |
| --- | --- | --- | --- | --- | --- | --- | --- | --- |
|  | median | q1 | q3 | median | q1 | q3 |  |  |
| Average urination frequency per day | 7 | 5 | 8 | 8 | 5.5 | 10.5 | -1.19 | 0.23 |
| Average urine volume per void | 170 | 125 | 215 | 200 | 103.5 | 270 | -0.18 | 0.86 |
| Urgency score | 1 | 0 | 2 | 1 | 0 | 2 | -0.04 | 0.97 |
| Average incontinence episodes | 0 | 0 | 2 | 0 | 0 | 0 | -2.31 | 0.02 |
| Average incontinence volume | 0 | 0 | 12 | 0 | 0 | 0 | -2.23 | 0.03 |
| VAS | 0 | 0 | 1 | 0 | 0 | 2 | -1.00 | 0.32 |
| QoL | 5 | 4 | 5 | 4 | 3 | 4 | -3.04 | 0.00 |
| OBASS | 2 | 1 | 5 | 2 | 1 | 4.5 | -0.69 | 0.49 |
| ICSI/ICPI | 6 | 1 | 9 | 5 | 0 | 9.5 | -0.39 | 0.70 |

Supplemental table 6. The comparison of technique details.

| Items | RP technique | OC technique |
| --- | --- | --- |
| Watient device | pulse generator +external programmer | pulse generator |
| Weight | about100g+150g | about 100g |
| Parameter settings | support | support |
| Setting electrode polarity, impedance | support | support |
| Battery life | about 1 week | about 2 weeks |
| Operating temperature | 10-40℃ | 10-40℃ |
| Operating humidity | 30-70% | 30-70% |
| Operating atmospheric pressure | 86-106kPa | 86-106kPa |
| Electromagnetic emission | GB 4824 group 2 category B | GB 4824 group 1 category A |
| Electromagnetic interference resistance | 3 A/m and 3 V/m | 3 A/m and 3 V/m |
| Requires network | require | not require |
| Patient self-operation steps | Schedule Programming；Log in and use the patient-side app，use external programmer | none |
| Does it require the patient to describe their own sensations? | require | require |
| Time required for a single programming | 20-40min | 10-30min |
| Physician operation steps | Log in and use the app for a period, programming | programming |
